# The Secretome of Endothelial Progenitor Cells Promotes Brain Endothelial Cell Activity through PI3-Kinase and MAP-Kinase

**DOI:** 10.1371/journal.pone.0095731

**Published:** 2014-04-22

**Authors:** Stefano Di Santo, Stefanie Seiler, Anna-Lena Fuchs, Jennifer Staudigl, Hans Rudolf Widmer

**Affiliations:** Department of Neurosurgery, Neurocenter and Regenerative Neuroscience Cluster, University of Bern, Inselspital, Bern, Switzerland; University of South Florida, United States of America

## Abstract

**Background:**

Angiogenesis and vascular remodelling are crucial events in tissue repair mechanisms promoted by cell transplantation. Current evidence underscores the importance of the soluble factors secreted by stem cells in tissue regeneration. In the present study we investigated the effects of paracrine factors derived from cultured endothelial progenitor cells (EPC) on rat brain endothelial cell properties and addressed the signaling pathways involved.

**Methods:**

Endothelial cells derived from rat brain (rBCEC4) were incubated with EPC-derived conditioned medium (EPC-CM). The angiogenic response of rBCEC4 to EPC-CM was assessed as effect on cell number, migration and tubular network formation. In addition, we have compared the outcome of the in vitro experiments with the effects on capillary sprouting from rat aortic rings. The specific PI3K/AKT inhibitor LY294002 and the MEK/ERK inhibitor PD98059 were used to study the involvement of these two signaling pathways in the transduction of the angiogenic effects of EPC-CM.

**Results:**

Viable cell number, migration and tubule network formation were significantly augmented upon incubation with EPC-CM. Similar findings were observed for aortic ring outgrowth with significantly longer sprouts. The EPC-CM-induced activities were significantly reduced by the blockage of the PI3K/AKT and MEK/ERK signaling pathways. Similarly to the outcome of the rBCEC4 experiments, inhibition of the PI3K/AKT and MEK/ERK pathways significantly interfered with capillary sprouting induced by EPC-CM.

**Conclusion:**

The present study demonstrates that EPC-derived paracrine factors substantially promote the angiogenic response of brain microvascular endothelial cells. In addition, our findings identified the PI3K/AKT and MEK/ERK pathways to play a central role in mediating these effects.

## Introduction

Extensive investigations are currently carried out to achieve replacement of damaged cells and tissue repair through cell transplantation [1]–[3]. Despite the experimental evidence of the beneficial use of stem and progenitor cells as therapeutic tools, their mechanisms of action are still not completely understood. Endothelial Progenitor Cells (EPC) hold great promise in the field of regenerative medicine. EPC are bone marrow-derived cells which play a critical role in the maintenance of endothelial homeostasis and contribute to physiological or pathological postnatal neovascularization. EPC are mobilized from the bone marrow into peripheral blood in response to chemoattractants released by ischemic or damaged tissues. Recent data suggest that the regenerative properties are not limited to vascularization and re-endothelization, but it is now evident that EPC can support tissue repair processes also in paradigms different form cardiovascular tissue as skin and neuronal tissue [4], [5]. In fact, there is compelling evidence that differentiation and incorporation into nascent vessels account only in part for the EPC actions and it is widely recognized that EPC-derived paracrine signals play a pivotal role in orchestrating the repair processes in damaged tissues [6], [7]. In line with this notion, considerable regenerative effects including increased neuronal plasticity have been detected after cell transplantation despite lack of neuronal differentiation [8]. Hence, soluble factors released from the graft are considered the key players in restorative cell transplantation approaches [9] and likely explain the apparent paradox in several paradigms of tissue regeneration with negligible engraftment of transplanted precursors into the host tissue [10]. We and others have previously reported that EPC secrete a large array of factors with immunomodulatory and angiogenic properties [11], [12]. In this context we have demonstrated that EPC-derived conditioned medium (EPC-CM) enhances mature endothelial cell viability [13]. Most importantly, the cocktail of trophic factors presented in EPC-CM has displayed remarkable therapeutic capacity comparable to cell transplantation in a model of hind limb ischemia in rats [11] and in a mouse stroke model [14]. A detailed description of EPC secretome has been reported [15] but the manner in which this complex mixture of factors modulates the activities of target cells at a molecular level remains elusive.

In endothelial cells PI3K/AKT and MEK/ERK signaling pathways play a primary role in the transduction of mitotic and pro-survival signals of many growth factors and cytokines (for review see [16], [17]). Angiogenesis and neurogenesis are coupled processes [18] and the intimate anatomical and physiological interaction of the nervous and vascular system pinpoints the importance of a functional vascular network for the maintenance of nervous tissue functions. As matter of fact, dysfunctions at the (micro)vascular level accompany neuronal degeneration in neurodegenerative disorders [19], [20]. As a corollary of these observations, it can be envisioned that interventions aimed to support endothelial functions and to promote revascularization not only might facilitate the restoration of appropriate tissue perfusion in cases of ischemia but also effectively enhance repair mechanisms in the nervous tissue [21].

In the present study we have investigated whether EPC-CM induces an angiogenic response in brain microvascular endothelial cells and assessed whether these effects might be mediated by PI3K/AKT and MEK/ERK pathways.

## Materials and Methods

### Endothelial cell culture

The rat brain microvascular cell line rBCEC4 was kindly provided by Dr. I. Blasig (Forschungsinstitut für Molekulare Pharmakologie, Berlin, Germany). rBCEC4 cells are derived by stable transfection and immortalization of rat brain microvascular endothelial cells with large T antigen of polyoma virus [22]. rBCEC4 cells show conserved properties of primary cells including an epitheloid morphology and the expression of endothelial and blood brain barrier markers. In addition, due to their formation of tight endothelial monolayers they are suitable for pharmacokinetic studies [22]. Cells were grown on 1% gelatin (Bio-Rad) coated plates in DMEM containing 5% Fetal Bovine Serum (FBS, Gibco), 1% antibiotics/antimycotics (Sigma), 1.2 mM glutamine (Invitrogen), 100 ug/ml heparin (Sigma) and 10 ug/ml endothelial cell growth factor (ECGF, Millipore), pH 7 in a humidified incubator at 37°C and 5% CO2.

### EPC culture and conditioned medium preparation

EPC were isolated from healthy donors peripheral blood using buffy coats purchased from the local Blood Bank (Blutspendedienst Bern) in accordance with previously published methods [23]. Mononuclear cell fraction was isolated by Ficoll gradient centrifugation using Histopaque (Sigma) and seeded on culture dishes coated with human fibronectin (10 µg/cm^2^, Sigma). The cultures were maintained in endothelial cell basal medium-2 (EBM-2) (Lonza) supplemented with endothelial growth medium SingleQuots and 5% FBS. After 4 days in culture, adherent cells were passaged and cultured through day 7 to obtain early outgrowth EPC. To produce EPC-CM, EPC were washed three times with PBS and incubated in growth factor-free EBM-2 containing 1% FBS for 48 hours under normoxic (21% O2, 5% CO2, 93.5% N2) or hypoxic conditions (1.5% O2, 5% CO2, 93.5% N2). A humidified gas-sorted anoxic incubator-gloved box (InVivo2 400, Ruskin) was used for hypoxic cultures. After incubation, the conditioned medium was collected and centrifuged at 10000 rpm at 4°C, the supernatant was sterile filtered and snap-frozen until use [11]. EBM-2 containing 1% FBS was treated in parallel to the cell cultures and served as control medium.

### Cytokine array

Presence of angiogenic growth factors in EPC-CM was screened using a Human Angiogenesis Antibody Array (RayBiotech) as previously described [13]. The chemiluminescent signal of each factor on the array was acquired by ChemiDoc XRS (Bio-Rad) and the intensity measured by densitometry analysis using ImageJ (http://rsbweb.nih.gov/ij/).

### Western Blot analysis

Two hundred thousand rBCEC4 grown in 6 well plates were incubated with control medium or EPC-CM for 15 min. The phosphorylation inhibitors [5 µM] LY294002 or [10 µM] PD98059 were administered 30 min prior and during exposure to EPC-CM. Following treatments, the cells were washed 3 times with cold HBSS and lysed in RIPA buffer (Pierce Biotechnology, Rockford, IL, USA) containing Protease Inhibitor Cocktail Set V (Calbiochem),1 µM Sodium Orthovanadate (Sigma), 10 µM PMSF (Sigma) and 1 µM Leupeptin (Sigma). After centrifugation the supernatant was collected and the protein concentration was estimated using the BCA kit (Pierce Biotechnology). Equal amount of proteins were resolved on a 12% polyacrylamide gel followed by overnight transfer and hybridization with primary antibodies: mouse anti total AKT (Cell Signaling), rabbit anti pAKT (Cell Signaling), mouse anti total ERK (Millipore), rabbit anti pERK (Millipore). As secondary antibodies Dylight-800 anti mouse and Dylight-680 anti rabbit were used. Immunoreactive bands were visualized using an Odissey CLx scanner (LI-COR) equipped with LI-COR Image. Quantification was performed on grey values converted images using the software ImageJ.

### Cell viability assay

Ten thousand rBCEC4 were seeded into 24 well plates and cultured as described above. Before treatment the cells were starved in EBM-2 containing 1% FBS for 24 hours. The cells were then exposed to EPC-CM in presence or absence of [5 µM] LY294002 and [10 µM] PD98059. After 24 hours the number of viable cells was assessed by the Presto Blue assay (Invitrogen) following the manufacturer's indications using a microplate fluorometer (Tecan Infinite 1000).

### Scratch Wound assay

Confluent rBCEC4 cultures were grown on 24 well plates, starved 8 hours in EBM2 medium containing 1% FBS. Thirty minutes before the EPC-CM treatment, cultures were incubated with [5 µM] LY294002 or [10 µM] PD98059 as indicated. Wounds were created by scratching the monolayer with a micropipette tip. Subsequently the cells were washed with PBS to remove not attached cells and incubated with the media according the assigned experimental group. Images were taken immediately after the scratch and 8 hours after the treatment with an inverted microscope (Nikon) equipped with a digital camera (Motic). The wound healing index was calculated by measuring the difference between the initial and final wound area using ImageJ software.

### In vitro tubular structure formation

The morphogenic capacity of rBCEC4 in vitro was assessed as previously described [24]. Melted growth factor reduced Matrigel (Becton Dickinson) was layered in 24 well plates and let to harden. Forty thousand cells were resuspended in control, as well as the other experimental culture media and seeded on the Matrigel layer. After 16 hours of incubation, the formation of endothelial cell tubular structure was assessed. Digital microphotographs were taken at low magnification with a light microscope from three randomly selected fields for data analysis. The angiogenic response of rBCEC4 was assessed measuring the total length, the number of branches and the area covered by the tubular structures with the aid of ImageJ software.

### Aortic ring assay

The sprouting from rat aortic rings was performed as previously described with minor modifications [11], [25], [26]. Animal experiments were performed in accordance with institutional guidelines and regulations. The procedures were approved by the Animal Research Ethics Committee of the Canton Berne Switzerland (permission number 85/10), and the University of Bern Animal Care and Use Committee, Switzerland. Briefly, adult female Wistar rats were anesthetized using an induction gas inhalation (4.5–5% Isoflurane, 75% N2O, 20% O2) followed by an i.p. injection with Ketamine (120 mg/kg body weight) and Xylazine (20 mg/kg body weight). In deep anesthesia the thoracic aorta was removed and then dissected free of perivascular adipose tissue, finally tissues were cut into 1 mm thick rings. Individual rings were placed in 24 well plates coated with growth factor reduced Matrigel (Becton Dickinson) and incubated with EPC-CM or control medium in presence or absence of the inhibitors. After 5 days of culture, digital microphotographs were taken from two randomly selected microscopic fields and analyzed with image J software. In each image, the angiogenic response was expressed as the total length of sprouts emerging from the aortic rings normalized for the circumference the aortic ring visualized in the image.

### Statistical analysis

For statistical analysis, a commercially available software package was used (Prism; GraphPad Software). One-way ANOVA followed by Tukey's multiple comparison test was used to compare group means, after testing for normality and equal variance of the data. Statistical significance was inferred at a 2-sided P ≤ 0.05. All experiments were performed in at least triplicates. Data are presented in percentage relative to the controls and values are given as mean ± standard error of the mean (s.e.m.).

## Results

### EPC-CM is composed of a mixture of angiogenic factors

**Figure 1:**
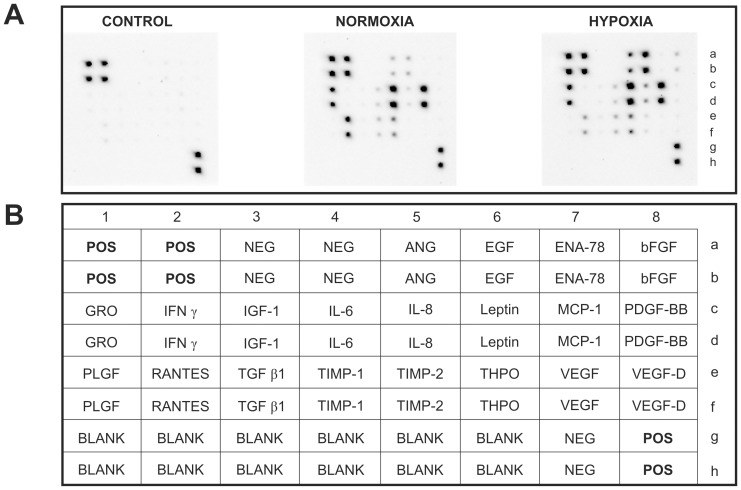
Cytokine Array. Presence of angiogenesis related factors in EPC-CM from normoxic (Normoxia) and hypoxic cultures (Hypoxia) was assessed by “Human Angiogenesis Antibody Array” **(A).** The identity of the specific antibodies spotted on the membrane is described in the table **(B).** Abbreviations: NEG: internal negative control; POS: internal positive control; ANG: angiogenin; ENA-78: epithelial-derived neutrophil-activating peptide 78; EGF: epidermal growth factor; bFGF: basic fibroblast growth factor; GRO: Growth-regulated alpha protein; IFN γ: interferon gamma; IL-6: interleukin-6; IL-8: interleukin-8; MCP-1: monocyte chemoattractant protein-1; PDGF-BB: platelet-derived growth factor beta; PLGF: placental growth factor; RANTES: regulated on activation, normal T cell expressed and secreted; TGF β1: Transforming growth factor beta 1; TIMP-1: tissue inhibitor of metalloproteinase-1; TIMP-2: tissue inhibitor of metalloproteinase-2; THPO: thrombopoietin; VEGF: vascular endothelial growth factor A; VEGF-D: vascular endothelial growth factor D.

We have used an antibody array to assess the composition of angiogenic growth factors in EPC-CM (Figure 1A) obtained from cultures incubated under standard or hypoxic conditions (1.5% oxygen). As expected, hypoxia increased the EPC-CM concentration of factors as angiogenin, EGF, bFGF, leptin, thrombopoietin, PDGF-BB, VEGF and VEGF-D. In contrast, RANTES was higher in the normoxic EPC-CM while other factors did not change in response to the oxygen level tested. Importantly, the analysis of the basal medium used as control revealed the presence of a very low basal level of angiogenic factors. For subsequent experiments only EPC-CM from hypoxic cultures was used.

### EPC-CM induces activation of PI3K and MAPK pathway in rBCEC4

**Figure 2:**
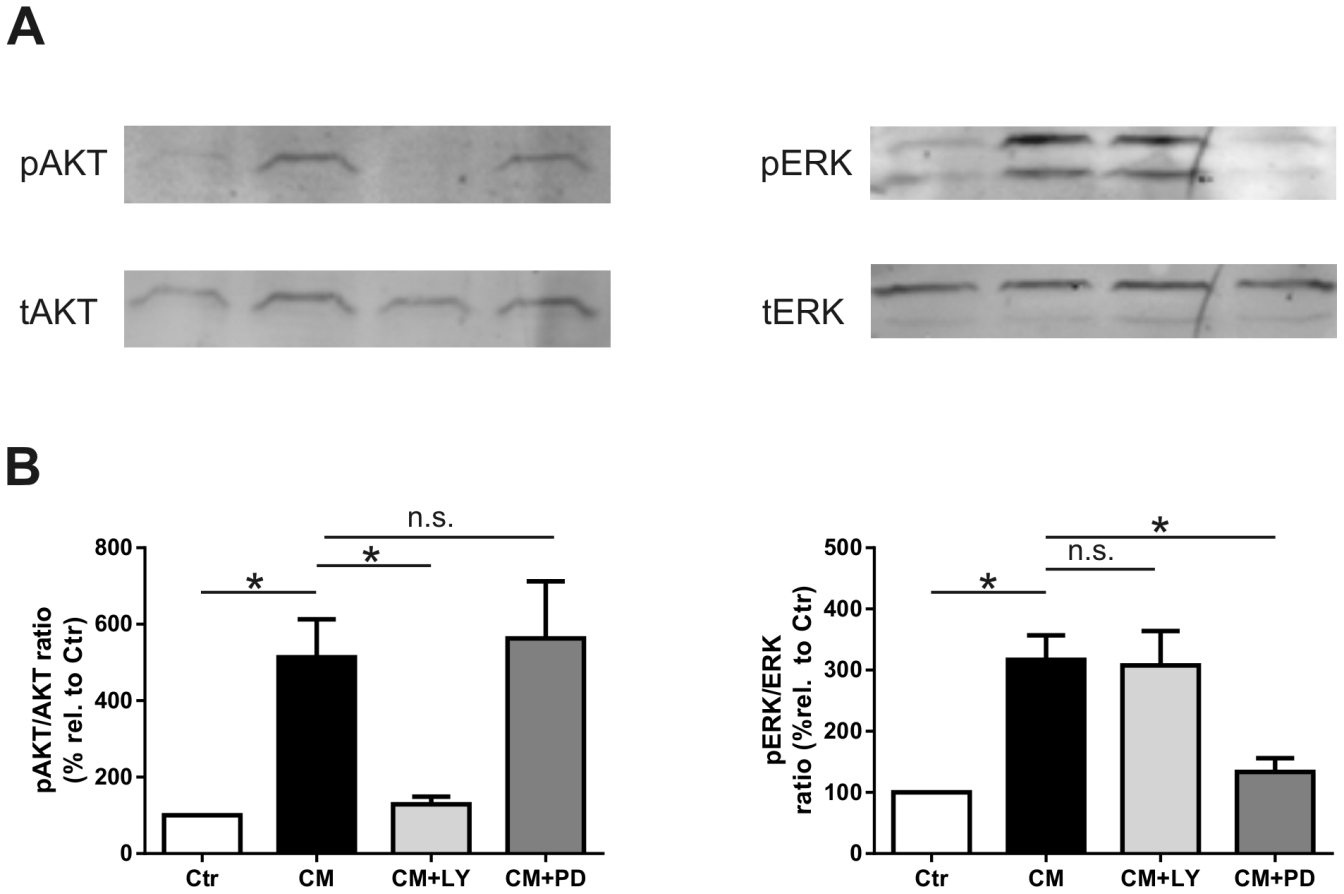
EPC-CM activates AKT and ERK pathways. Representative immunoblots **(A)** and quantitative analysis **(B)** of rBCEC4 lysates incubated with vehicle (Ctr) or EPC-CM (CM) in presence of either LY294002 (CM+LY) or PD98509 (CM+PD). Exposure of rBCEC4 to EPC-CM resulted in a marked increase of both phosphorylated AKT and phosphorylated ERK (pAKT and pERK) with regards to the total AKT and ERK levels (tAKT and tERK). Concomitant incubation with EPC-CM and the corresponding inhibitors almost completely blocked the phosphorylation of AKT and ERK. Data are given as mean + s.e.m. and values are presented as percentage of corresponding controls. *: p < 0.05.

We next reasoned whether the factors present in EPC-CM could induce PI3K/AKT and MEK/ERK pathways activation in brain microvascular endothelial cells. Protein lysates of rBCEC4 cultures treated with EPC-CM disclosed a time–dependent activation of PI3K/AKT and MEK/ERK pathways with AKT and ERK phosphorylation peaking at 15 minutes of incubation (Fig. S1). Quantitative analysis of AKT and ERK phoshorylation at this time point displayed a robust increase in rBCEC4 incubated with EPC-CM (513.5 ± 99.4% and 317 ± 39.8% band intensities relative to controls, for pAKT and pERK, respectively). As expected, this effect was abrogated by co-incubation with the respective inhibitors (to 129.1 ± 20.1% and 133.5 ± 22.7% compared to controls, for pAKT and pERK, respectively) (Fig. 2).

### rBCEC4 basal functions are not affected by PI3K/AKT or MEK/ERK inhibition

**Figure 3:**
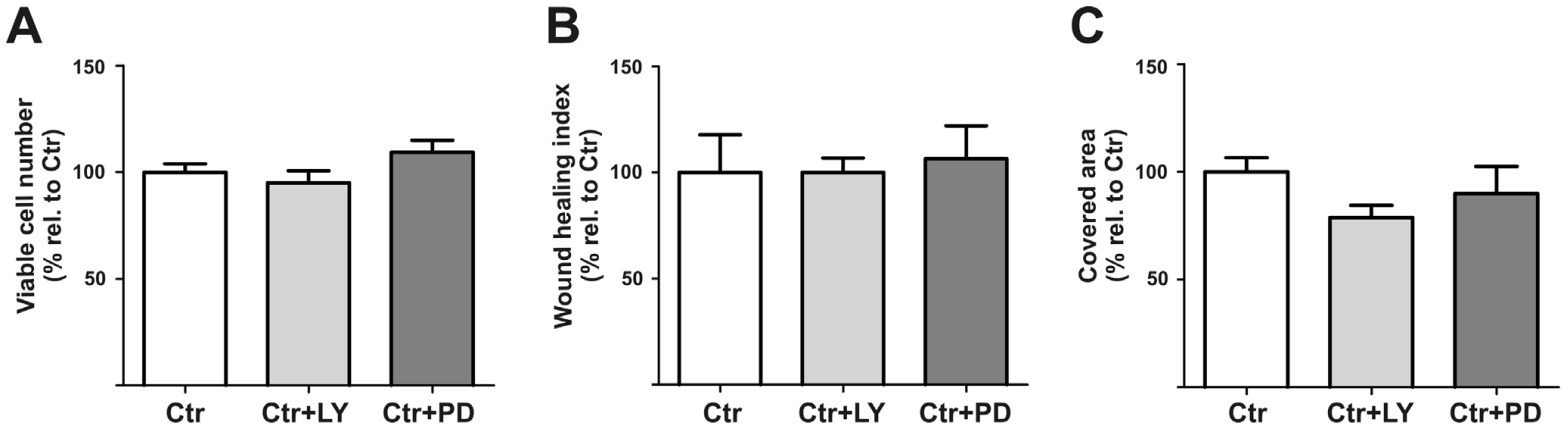
Basal functions of rBCEC4 are not altered by LY294002 or PD98059. Viability (A), migration (B) and capacity to organize in tubular structures (C) by rBCEC4 was not significantly changed compared to controls (Ctr) when incubated in presence of [5 µM] LY294002 or [10 µM] PD98059. Data are given as mean + s.e.m. and values are presented as percentage of corresponding controls.

We have conducted preliminary experiments in order to assess whether the treatments with the PI3K/AKT or MEK/ERK inhibitors would affect the basal functions of rBCEC4 under control conditions (Fig. 3). Incubation of rBCEC4 in presence of [5 µM] LY294002 or [10 µM] PD98059 did not significantly alter the viability (Fig. 3A), capacity to migrate (Fig. 3B) and form tubular structures on Matrigel (Fig. 3C).

### EPC-CM induces rBCEC4 viability

**Figure 4:**
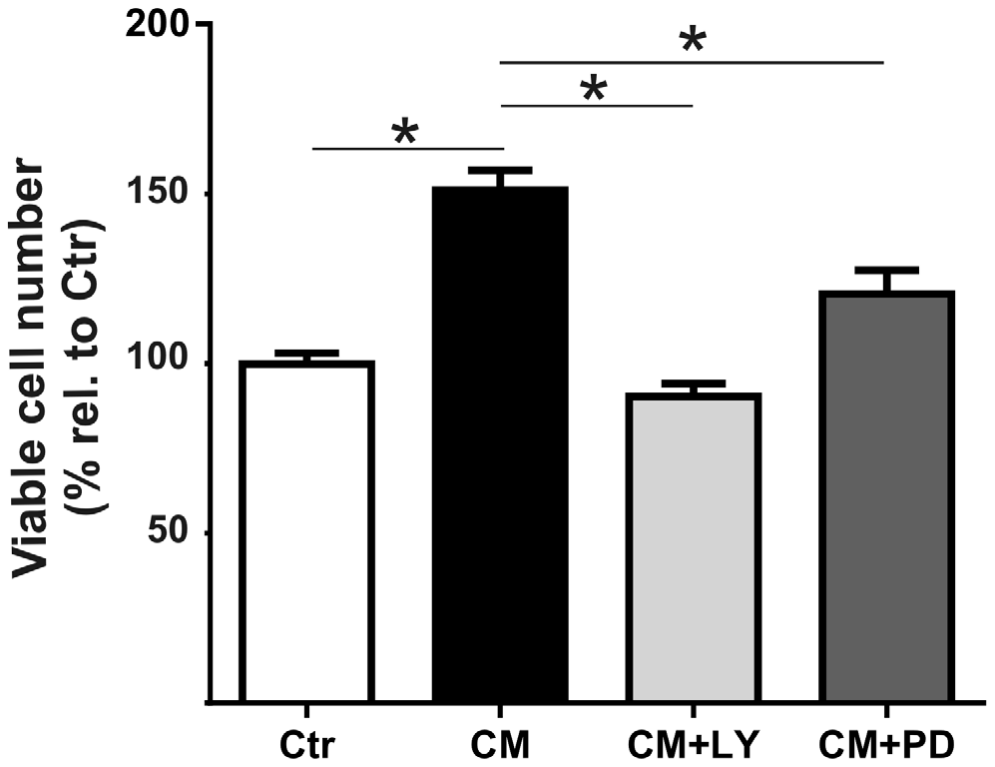
EPC-CM-induced rBCEC4 viability is AKT and ERK dependent. rBCEC4 incubated with EPC-CM (CM) displayed higher viable cell numbers assessed with the Presto Blue assay as compared to control (Ctr). The EPC-CM induced higher viable cell number was inhibited by AKT (CM+LY) as well as ERK (CM+PD) phosphorylation inhibitors ([5 µM] and [10 µM], respectively). Data are given as mean + s.e.m. and values are presented as percentage of control. *: p < 0.05.

To examine whether soluble factors secreted by EPCs promote endothelial cell viability, rBCEC4 were incubated with EPC-CM. Administration of EPC-CM resulted in a significant increase of viable cell numbers as compared to controls (to 150.9 ± 6.0%). In order to assess whether the effect on rBCEC4 viability is mediated by AKT and/or ERK pathway, EPC-CM incubation was carried out in absence or presence of the PI3K/AKT or MEK/ERK inhibitors. Inhibition of the PI3K/AKT pathway with [5 µM] LY294002 significantly reduced EPC-CM dependent cell number raise (to 90.2 ± 3.8%). Similarly, but to a minor extent, inhibition of the MEK/ERK pathway with [10 µM] PD98059 resulted in significantly lower viable cell numbers (to 120.5 ± 6.9%) (Fig. 4).

### EPC-CM supports wound closure through PI3K/AKT and MEK/ERK pathway

**Figure 5:**
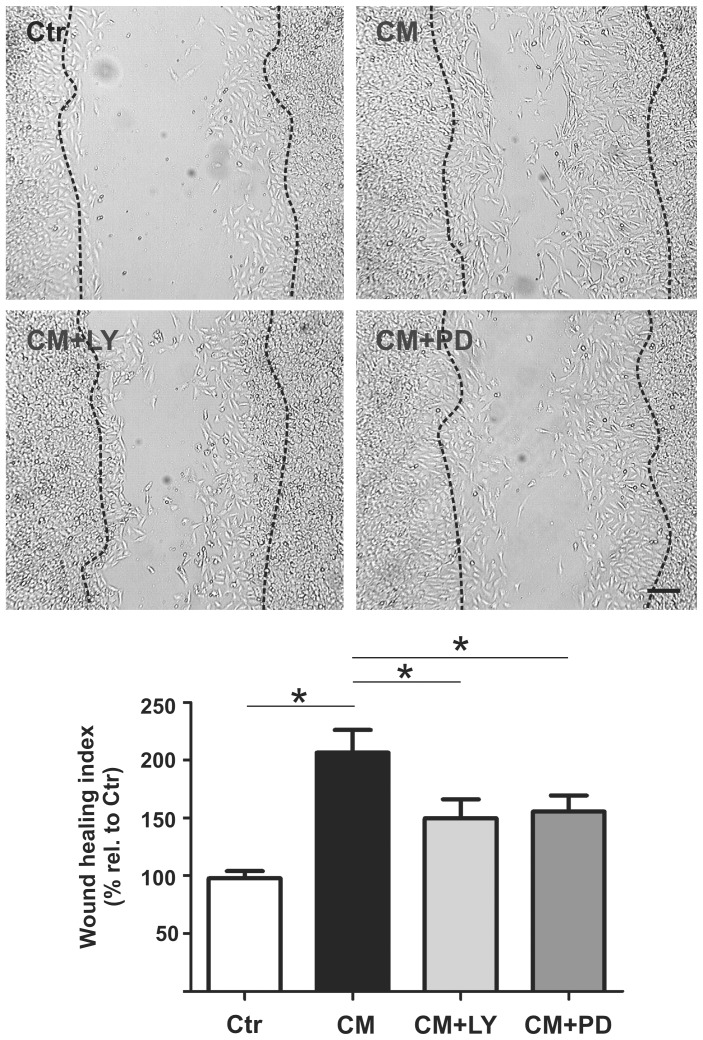
EPC-CM-induced rBCEC4 migration is AKT and ERK dependent. Representative microphotographs (upper panels) and quantitative analysis (lower panel) of rBCEC4 wound healing capacity. rBCEC4 incubated with EPC-CM (CM) displayed higher migration rate in the wound healing-scratch test as compared to control (Ctr). Scale bar: 100 µm. EPC-CM induced migration was inhibited by AKT (CM+LY) as well as ERK (CM+PD) phosphorylation inhibitors ([5 µM] and [10 µM], respectively). Data are given as mean + s.e.m. and values are presented as percentage of control. *: p < 0.05.

The capacity of EPC-CM to enhance cell migration was assessed by a scratch wound assay. Cultures incubated with EPC-CM displayed substantially accelerated wound closure compared to control (by 206.0 ± 19.7%). Treatment with the PI3K/AKT or MEK/ERK pathways inhibitors ([5 µM] and [10 µM], respectively) significantly decreased but did not completely block the EPC-CM-induced migration (to 149.7 ± 16.4% and 155.0 ± 14.1% relative to control group, respectively) (Fig. 5).

### EPC-CM promotes tube formation through PI3K/AKT and MEK/ERK activation

**Figure 6:**
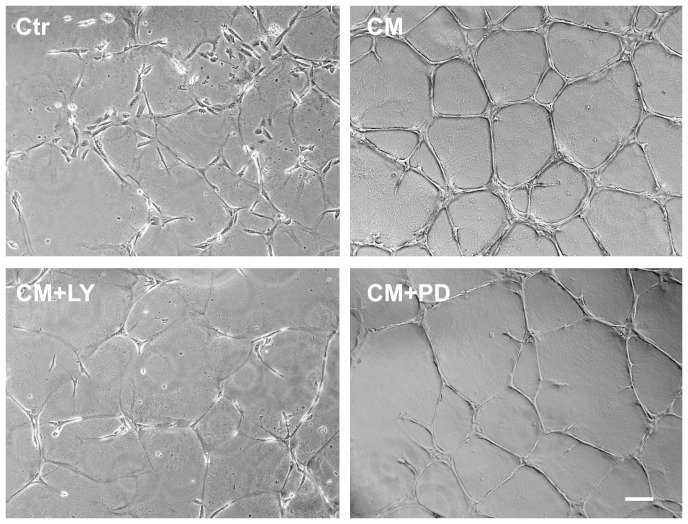
Angiogenic potential of EPC-CM on tubular structure formation. Representative microphotographs of tubular network formation by microvascular endothelial cells on growth factor reduced Matrigel. EPC-CM incubation (CM) strikingly promoted the tube-like structure formation as compared to control medium (Ctr). Supplementation of EPC-CM with the inhibitor of AKT phosphorylation (CM+LY) ([5 µM]) substantially attenuated EPC-CM induced tube formation. Similarly, addition of the inhibitor of ERK phosphorylation (CM+PD) ([10 µM]) resulted in a decrease of tube formation, however, to a lesser degree as compared to the CM+LY group. Scale bar: 100 µm.

**Figure 7:**
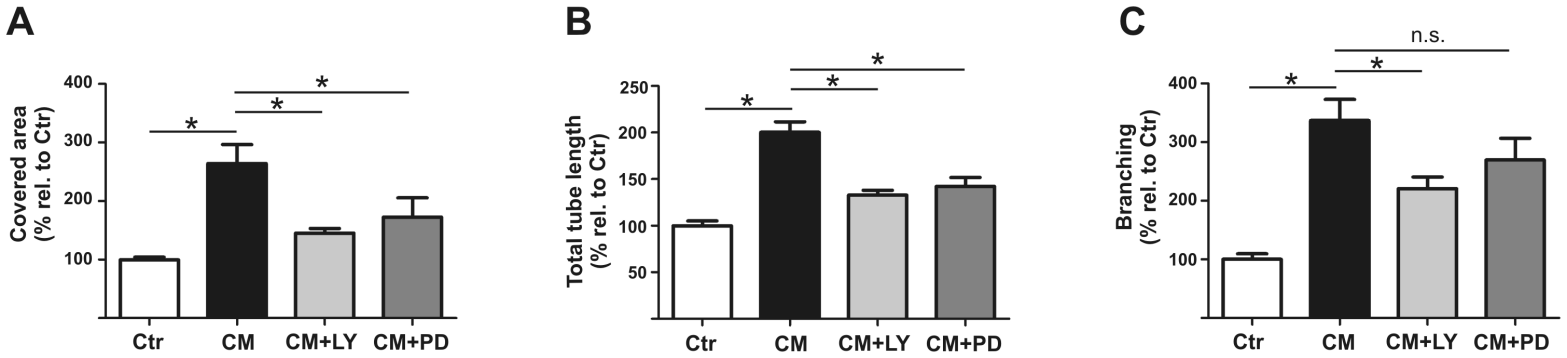
EPC-CM effects on tubular structure formation are AKT and ERK dependent. Quantitative analysis of tubular network formation by microvascular endothelial cells on growth factor reduced Matrigel. EPC-CM incubation (CM) significantly promoted total area covered by the tube-like structures (A), total sprout length (B) and branching (C) as compared to control medium (Ctr). Supplementation of EPC-CM with the inhibitor of AKT phosphorylation (CM+LY) ([5 µM]) substantially attenuated all three parameters while the addition of the inhibitor of ERK phosphorylation (CM+PD) ([10 µM]) resulted in a significant decrease of covered area and total tube length but had no significant effect on the reduction of the number of branches. Data are given as mean + s.e.m. and values are presented as percentage of corresponding controls. *: p < 0.05.

EPC-CM considerably enhanced the capacity of rBCEC4 to form tubular structures on Matrigel. These EPC-CM-dependent morphogenic properties were clearly diminished by inhibition of the PI3K/AKT and the MEK/ERK pathway (Fig. 6, 7). Quantitative analyzes revealed that the incubation with EPC-CM resulted in significantly wider total area covered by capillary-like structures (2.6 fold), total tubule length (2 fold) and increased number of branches (3.3 fold) as compared to control medium. Concomitant treatment with [5 µM] LY294002 significantly attenuated the total area covered by capillary-like structures, total tubular length and number of branches induced by EPC-CM. Similarly, the presence of [10 µM] PD98059 significantly reduced the total area covered by capillary-like structures and total tubular length induced by EPC-CM. Notably, inhibition of the MEK/ERK pathway attenuated number of branches induced by EPC-CM but the difference did not reach statistical significance (Fig. 6, 7).

### EPC-CM supports angiogenesis through PI3K/AKT and MEK/ERK activation

Based on the outcome of the PI3K/AKT and MEK/ERK pathway inhibition experiments using the rBCEC4 cell line we reasoned whether similar effects could be reproduced on tissue explants. Hence, we used the well established aortic ring assay to monitor the involvement of the PI3K/AKT and MEK/ERK pathways on EPC-CM induced angiogenesis.

**Figure 8:**
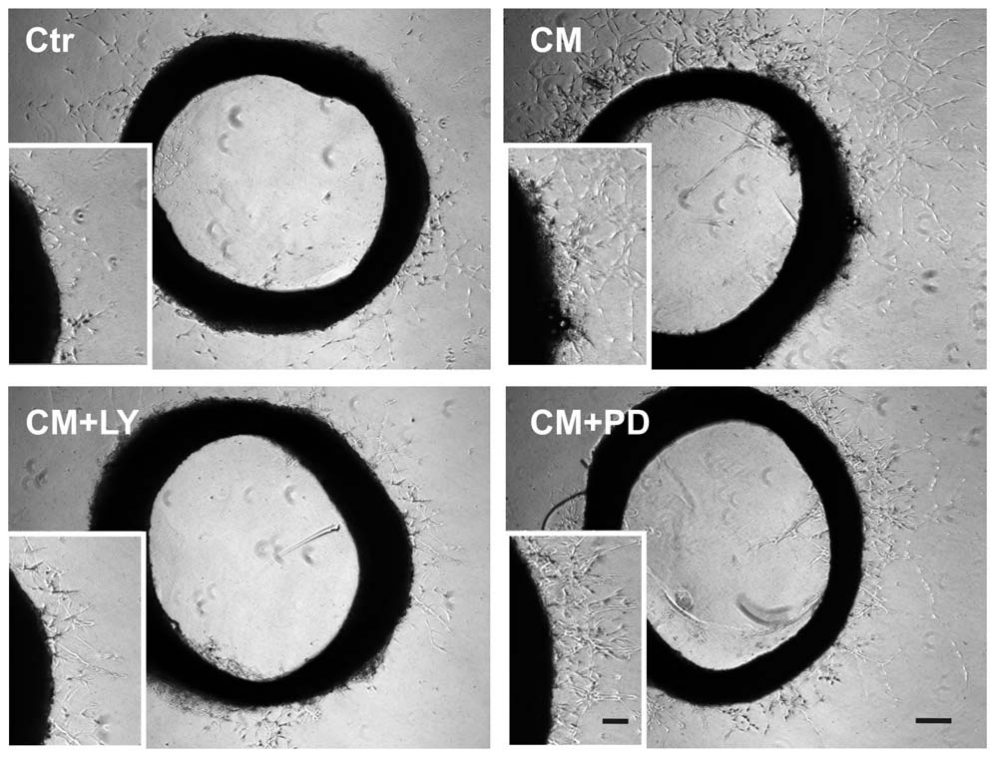
Angiogenic potential of EPC-CM on *ex vivo* aortic rings. Representative microphotographs of vascular outgrowth from rat aortic rings embedded in growth factor reduced-Matrigel. Incubation with EPC-CM (CM) enhanced the formation of vascular outgrowth from the aortic rings compared to control medium incubation (Ctr). EPC-CM-enhanced capillary outgrowth was abolished by the addition of [5 µM] LY294002 (CM+LY). Similarly, incubation with EPC-CM in presence of [10 µM] PD98509 (CM+PD) significantly impaired total sprout length induced by EPC-CM. Scale bars: 200 µm and 100 µm (inserts).

**Figure 9:**
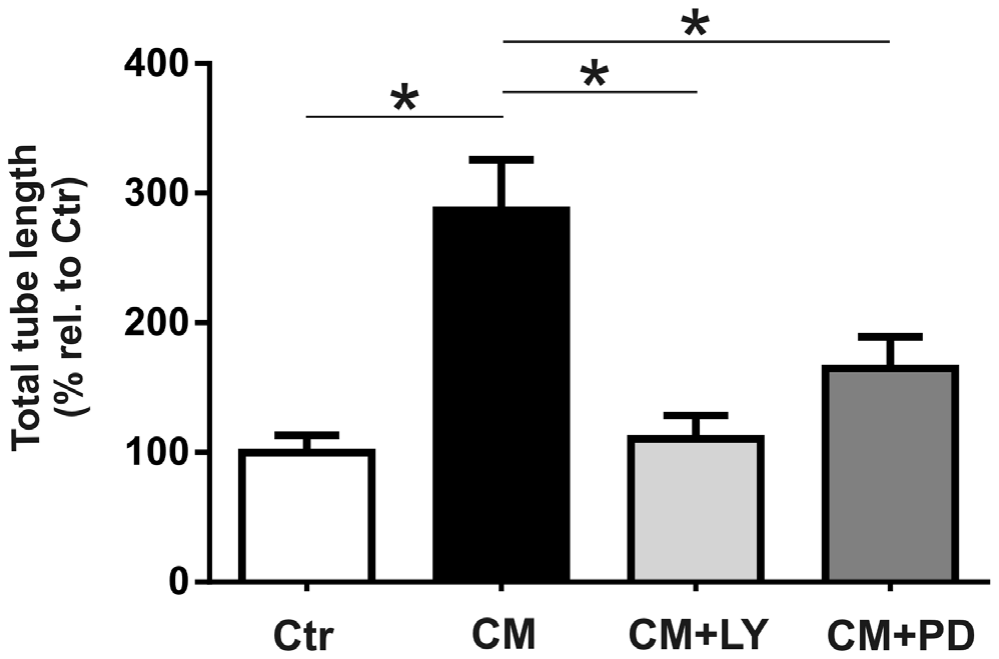
EPC-CM effects on total sprout length of *ex vivo* aortic rings are AKT and ERK dependent. Quantitative analysis of vascular outgrowth from rat aortic rings embedded in growth factor reduced-Matrigel. Incubation with EPC-CM (CM) enhanced total sprout length from the aortic rings compared to control medium incubation (Ctr). This effect was significantly abolished by addition of [5 µM] LY294002 (CM+LY) as well as of [10 µM] PD98509 (CM+PD). Data are given as mean + s.e.m. and values are presented as percentage of control. *: p < 0.05.

Accordingly with previously published data [11], length of sprouts emerging from the aortic wall was significantly higher in the EPC-CM treated group (2.9 fold) as compared to the control. This pro-angiogenic effect of EPC-CM was completely abrogated by the presence of the PI3K/AKT inhibitor ([5 µM]) and significantly attenuated by the supplementation of the MEK/ERK inhibitor ([10 µM]) (Fig. 8, 9).

## Discussion

EPC contribute to the maintenance of the endothelial integrity and the vascular growth. For these relevant characteristics, EPC are considered a promising therapeutic tool for vascular medicine and tissue regeneration [27]. There is now compelling evidence that the cytoprotective and angiogenic properties of EPC substantially rely on paracrine mechanisms [28]–[30]. We have previously demonstrated that soluble factors secreted by EPC induce a protective response in cultured endothelial cells [13] and display potent angiogenic effects in an animal model of chronic muscle ischemia [11]. The present study further extends these observations and provides important clues on the mechanisms of action underlying the effects of the soluble factors secreted by EPC. Specifically, we report that in the brain microvascular endothelial cell line rBCEC4 the PI3K/AKT and the MEK/ERK pathways play a fundamental role in transducing the angiogenic cues conveyed by the EPC-paracrine factors.

The identification of the array of factors secreted by EPC is still object of intense investigation [12], [15], [31]. We and others have previously documented the presence of several growth factors and cytokines in EPC-CM [7], [11] and in the present work we consistently report the increased level of angiogenic factors in EPC-CM as result of hypoxic conditioning. Overall, EPC-CM has been found to promote survival, morphogenesis and migration of endothelial cells [13], [32]. At the intracellular signaling level, it is conceivable that these cellular responses result from concomitant activation of different pathways in turn induced by the plethora of factors contained in EPC-CM. Indeed, factors secreted from EPC into the medium like VEGF and EGF among many others can activate AKT and ERK pathways which are pivotal signaling steps in angiogenesis [33], [34]. In line with this observation and consistently with the data reported here, Xia and colleagues described that the activation of AKT as well as of ERK by EPC soluble factors resulted in increased proliferation and reduced apoptosis of lung microvascular endothelial cells [35]. Likewise, it has been shown that AKT activation by umbilical cord monocyte-derived paracrine factors is indispensable to confer protection against coronary artery endothelial cell apoptosis [36].

In the frame of studies addressing the paracrine signals, great emphasis is usually given to growth factors and cytokines. Indeed, the successful identification of the key element of stem/progenitor cells secretome has been reported [37], [38] while our previous findings suggest that synergic action of multiple factors might also account for the effects of EPC-CM [13], [24]. However, it should be kept in mind that proteins are not the sole constituents of conditioned media. Hence, in the present study it cannot be excluded that non-proteiceous compounds might participate at least in part in influencing the endothelial cell activity reported here. Among the different molecules that can be found in the conditioned medium, the compounds of the cellular metabolism as citrate [39], ATP [40] or 20-Hydroxyeicosatetraenoic acid (20-HETE, an eicosanoid) [41] have been shown to promote angiogenesis directly activating AKT and ERK or synergizing growth factors action. Hence, the dissection of conditioned medium components and signaling pathway activation in target cells can be considered the two sides of the same coin.

The capacity to elicit angiogenic and cytoprotective actions through paracrine factors is not a unique feature of EPC. In fact, it has been reported that conditioned medium from mononuclear cells [42], [43], embryonic stem cells [44], mesenchymal stem cells [45]–[49] promoted vascular growth and endothelial cell as well as cardiomyocytes survival and migration. Noteworthy, these secretome-induced actions involved primarily the activation of the PI3K/AKT pathway [44], [45]. On the other hand, the MEK/ERK pathway, despite induction, seems to play a less relevant role in driving the angiogenic program promoted by paracrine factors [45]. These findings are overall in line with the results reported here. Specifically, in our experimental settings we observed that EPC-CM promoted all the angiogenic activities tested and involved AKT activation. Differently, even though the ERK pathway was significantly involved, it appears to have a slightly smaller impact particularly on viable cell numbers and on capillary sprouting in the aortic ring assay. It is likely that the different outcome in terms of viable endothelial cells resulting from AKT or ERK inhibition accounts, at least in part, for the shorter sprout length observed in the aortic ring assay. More studies are needed to define the responses of the different cell types present in the aortic wall to EPC-CM including fibroblast, mural cells and macrophages [50].

The angiogenic properties of EPC-CM on rBCEC4 reported here are consistent with previous works employing endothelial cells of different origins. In these studies a similar response of to EPC-CM with regards to proliferation, migration and capillary network formation in vitro has been outlined [13], [32], [35], [36]. Current data suggest that the effects of EPC-CM might go beyond survival of local endothelial cells. In fact it has been reported that AKT and ERK activated endothelial cells in bone marrow differently modulate hematopoietic stem cells self-renewal and differentiation [51]. It is thus possible that a bidirectional crosstalk between endothelial cells and EPC through soluble factors might take place also in the periphery of the circulatory system and have a relevant role in tissue homeostasis.

It is clear that the restoration of appropriate perfusion and brain blood vessels functionality are essential to treat conditions like ischemic stroke. Noteworthy, several lines of evidence suggest that vascular impairment plays a substantial role in the pathogenesis of neurodegenerative disorders like Alzheimer and Parkinson diseases [19], [20]. Thus, interventions targeting the endothelium might offer new therapeutic options for neuronal repair in a wide range of diseases. In the present study a rat cell line was deliberately chosen to assess the response of EPC-CM of human origin in view of future application of EPC-CM in rat models of neurodegenerative conditions. It has been reported that rBCEC4 cells preserve many properties of primary cells and are phenotypically stable [22] and are useful tools for studies involving interactions between the endothelium and the surrounding neuronal tissue [52]. The present work supports the concept of a therapeutic approach based on EPC-derived secretome for the treatment of conditions involving endothelial dysfunction and to the best of our knowledge it is the first study addressing the effect of factors secreted by EPC on brain microvascular endothelial cells.

In summary, the present study adds further evidence of the angiogenic potential of EPC-CM and provides the basis of understanding mechanisms of action of EPC by paracrine secretion.

## Supporting Information

Figure S1 Time course of AKT and ERK phosphorylation. Representative immunoblots of the time course of AKT and ERK phosphorylation in rBCEC4 cells treated with EPC-CM. The level of AKT and ERK phosphorylation peaks at 15 minutes (15′) of incubation with EPC-CM and gradually declines to the control (Ctr) level after 16 hours (16 hr).
(TIF)
