# Supplementary figures and images for: The Secretome of Endothelial Progenitor Cells Promotes Brain Endothelial Cell Activity through PI3-Kinase and MAP-Kinase
Source: PLoS One. 2014 Apr 22;9(4):e95731. doi: 10.1371/journal.pone.0095731 (PMC3995762; doi:10.1371/journal.pone.0095731)

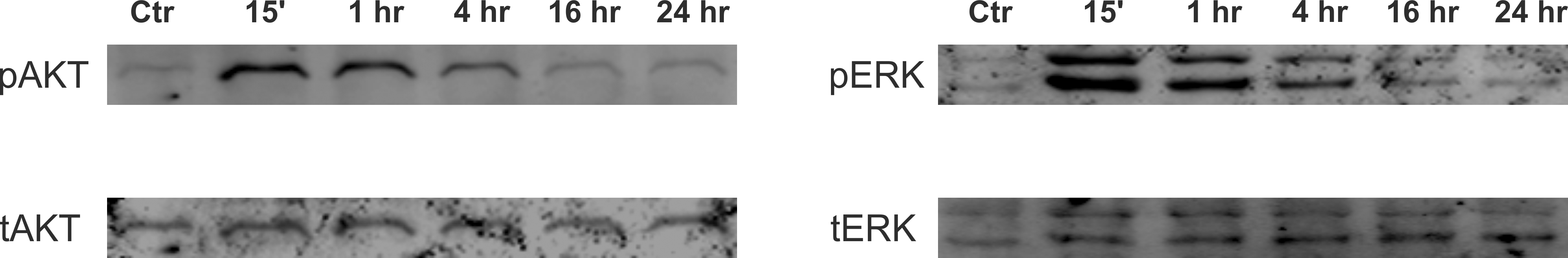

Supplement: Figure S1 — Time course of AKT and ERK phosphorylation. Representative immunoblots of the time course of AKT and ERK phosphorylation in rBCEC4 cells treated with EPC-CM. The level of AKT and ERK phosphorylation peaks at 15 minutes (15′) of incubation with EPC-CM and gradually declines to the control (Ctr) level after 16 hours (16 hr). (TIF) [file pone.0095731.s001.tif]
